# Capsule Independent Uptake of the Fungal Pathogen *Cryptococcus neoformans* into Brain Microvascular Endothelial Cells

**DOI:** 10.1371/journal.pone.0035455

**Published:** 2012-04-17

**Authors:** Wilber Sabiiti, Robin C. May

**Affiliations:** Institute of Microbiology & Infection, School of Biosciences, College of Life and Environmental Sciences, University of Birmingham, Birmingham, United Kingdom; University of Minnesota, United States of America

## Abstract

Cryptococcosis is a life-threatening fungal disease with a high rate of mortality among HIV/AIDS patients across the world. The ability to penetrate the blood-brain barrier (BBB) is central to the pathogenesis of cryptococcosis, but the way in which this occurs remains unclear. Here we use both mouse and human brain derived endothelial cells (bEnd3 and hCMEC/D3) to accurately quantify fungal uptake and survival within brain endothelial cells. Our data indicate that the adherence and internalisation of cryptococci by brain microvascular endothelial cells is an infrequent event involving small numbers of cryptococcal yeast cells. Interestingly, this process requires neither active signalling from the fungus nor the presence of the fungal capsule. Thus entry into brain microvascular endothelial cells is most likely a passive event that occurs following ‘trapping’ within capillary beds of the BBB.

## Introduction

Cryptococcosis is a life-threatening disease caused primarily by the human fungal pathogen *Cryptococcus neoformans*. Cryptococcal infection can potentially occur in any part of the human body (reviewed in [1]), although central nervous system (CNS) cryptococcosis accounts for most clinical presentation. Globally, fatalities due to cryptococcal meningitis were recently estimated at over 600,000 cases per year of which 504000 (81%) occur in Sub-Saharan Africa [2]. The victims of cryptococcal infection are predominantly immunocompromised people infected with *C. neoformans*, although there is an increasing incidence of immunocompetent cryptococcosis caused by *C. gattii* [3]–[7]. HIV/AIDS is the major predisposing condition for cryptococcosis with 10–15% HIV patients acquiring cryptococcal infection, although prolonged glucocorticosteroid therapy and solid organ transplantation are increasingly becoming important [8], [9].

The route of cryptococcal infection is believed to be through inhalation of airborne basidiospores or desiccated yeast cells from an environmental source to the lungs. The fungal yeast cells may stay dormant in the host, and can potentially disseminate to all body organs but there is a high propensity of dissemination to the brain [10]. Once in the brain the fungus causes meningoencephalitis, a severe form of the disease, which is uniformly fatal if untreated [11]. Even with the most effective antifungal therapy, the fatality rate remains high in HIV-associated crytococcosis (10–25% and >40% in rich and poor settings respectively [12]–[17] Dissemination to the brain requires that *C. neoformans* penetrate the normally impermeable blood-brain barrier (BBB) [18]. The BBB is made of microvascular endothelial cells supported by astrocytic foot processes, pericytes and neuronal processes [19]. Brain microvascular endothelial cells form strong tight junctions, which present a formidable barrier to any invading pathogens [18]–[20]. The mechanism by which *C. neoformans* penetrates this barrier is not currently understood, although several possibilities have been proposed, including passage between neighbouring endothelial cells (paracellular entry), carriage into the CNS within infected phagocytes (Trojan Horse model), or uptake by and traversal through endothelial cells (transcytosis) [21], [22]. In the transcellular model of traversal, adherence to and uptake of cryptococci by brain microvascular endothelial cells (BMEC) must occur before transit into the brain. In support of this model, Chang et al used electron microscopy to demonstrate that cryptococcal yeast cells could adhere to and become internalised by brain microvascular endothelial cells [23].

Several pathogen-generated microbial factors including urease, laccase, capsule and hyaluronic acid have been implicated in modulating the *Cryptococcus* – blood-brain barrier interaction [24], [25]. The capsule is a major virulence factor and its role in pathogen – phagocyte interaction and systemic dissemination of *Cryptococcus* is well documented [26]. However, the role of capsule in regulating CNS invasion remains unclear. Capsule associated structural changes such as phenotypic switching (rough to smooth) have been reported to enhance crossing of the blood-brain barrier [27]–[31], but a recent study using intravital real time imaging demonstrated that encapsulated and acapsular strains of *C. neoformans* had an equal ability to associate with – and transmigrate across - the microvascular endothelium into the brain [32]. Despite these recent advances, however, there are currently no quantitative data on cryptococcal uptake by brain endothelial cells in the presence and absence of capsule. Here we report the first attempts to address this, by using an *in vitro* brain endothelial cell culture to quantify association and uptake of cryptococci.

## Materials and Methods

### Yeast culture

Two sets of isogenic *C. neoformans* strains, serotype A H99 and its isogenic acapsular strain cap59 and serotype D B3501 with its isogenic acapsular strain B4131 were used. Strains were propagated on YPD agar (1% yeast extract, 1% peptone, 2% dextrose and 1% agar) at 25°C. Prior to experimentation, cultures of both strains were grown in YPD broth (1% yeast extract, 1% peptone and 2% dextrose) at 25°C with rotation at 20 RPM overnight. The yeast cells were washed with sterile phosphate buffered saline (PBS) and stained with 0.5 mg/ml FITC for 30 min with shaking (Labrolller, Labnet Inc.) at room temperature. The required infection inoculum (of 2×10^6^ yeast cells) was determined by counting using a haemocytometer.

### Tissue culture

Two types of brain microvascular endothelial cell-lines, the immortalized mouse brain derived endothelial (bEnd3) cells and the human brain capillary microvascular endothelial cells (hCMEC/D3) were used. The bEnd3 cells were grown to monolayer confluence in 24 well tissue culture plates (Greiner, UK) containing Dulbecco's modified Eagle's medium (DMEM, Sigma Aldrich) supplemented with 10% foetal bovine serum (FBS), I% streptomycin/penicillin and 2 mM L-glutamine, 1% non-essential aminoacids, 1% Sodium pyruvate and 5 µM 2-Mercaptoethanol. HCMEC/D3 cells were grown in endothelial growth medium 2 (EGM-2, Lonza, UK) in 24 well tissue culture plates precoated with Calf Skin collagen (Sigma UK). Seeding plates with 10^5^ endothelial cells per well, ensured even growth of a cell monolayer. The culture was maintained at 37°C with 5%CO_2_ for 4–6 days to obtain a fully matured cell monolayer. For microscopic examination, 13 mm sterile glass coverslips (collagen coated for hCMEC/D3 cells) were inserted into the 24 well plates before seeding with endothelial cells, allowing the monolayer to grow on the coverslip, which could then be easily transferred for microscopy. Prior to infection, tissue culture growth medium was replaced with serum free medium and incubated for 1 hr at 37°C. The cultures were then inoculated with 2×10^6^ yeast cells per well, producing an approximate infection ratio of 1: 3 (target: effector). Infections were allowed to proceed for either 2 hr or 4 hrs, as described, at 37°C with 5% CO_2_. To ensure that the infection media did not have a negative effect on cryptococcal growth, cryptococci (10^5^ yeast cells/ml) were directly inoculated into bEnd3 and or hCMEC/D3 infection medium and growth recorded over 24 hrs.

### Quantification of yeast cell association with, and internalization by, BMEC cells

The rate of cryptococcal yeast cell association with brain microvascular endothelial cells (BMEC), bEnd3 and hCMEC/D3 was determined both microscopically and using CFU counts. In the former, dual colour fluorescence microscopy (Nikon Eclipse T*i* - S, Japan) was used to determine associated (adherent and internalized) yeast cells. After infection, the non-adherent yeast cells were removed by washing four times with sterile PBS and the extracellular adherent yeast cells stained for 10–15 min with 20 µg/ml calcoflour white (adapted from [33]) at room temperature. Since mammalian cells are impermeable to calcofluor white, internalized yeast retained the green FITC signal while adherent cells stained blue. Nine random fields per coverslip were viewed and the internalized and non-internalized yeasts therein counted. Association (total number of yeast cells –attached or internalized by BMEC) and internalization were determined and compared to the original inoculum.

Since this microscopic approach did not distinguish between live and dead cryptococci, we exploited colony forming unit (CFU) assays to determine the viability of cryptococci that had been internalised by cells of the BBB. After removal of non-adherent cryptococci by extensive washing, endothelial cells were lysed with 200 µl sterile water for 15 min at 37°C to release internalized cryptococci and the lysate plated on YPD agar for colony counts (CFU assay). The associated cryptococci were determined as the ratio of cryptococcal yeast cells (CFU/ml) to the original inoculum.

Thus, by comparing data derived both from microscopy (which distinguishes internalised from attached cryptococci, but not live from dead cells) and from CFU counts (which have the opposite profile) we were able to accurately estimate both uptake and survival of cryptococci. The results were recorded as endothelial cell associated cryptococci per well, which is equivalent to cryptococci per coverslip for microscopy and CFU/ml for colony counts.

### Opsonisation

Antibodies to *Cryptococcus* have been reported to exist in circulation as early as childhood [34]. Furthermore, phagocytosis studies using macrophages have shown that internalization of cryptococcal yeast cells is enhanced by antibody and or complement mediated opsonisation [35]–[37]. However, no studies have investigated whether adherence and uptake of cryptococci by BMEC requires opsonisation. To address this, opsonised and non-oposonized live and heat killed H99 cryptococci were incubated with BMEC for 2 hr and 4 hr at 37°C. Opsonisation was done by adding 5 µg/ml the capsule specific 18B7 mouse IgG (a kind gift from Arturo Casadevall) to 200 µl aliquot of yeast cells and rotated (Labroller, Labnet Inc, US) at room temperature for 30 min prior to infection. The rate of association and internalization was determined as described above.

### Role of viability in *Cryptococcus* – BMEC association and internalization

We tested whether dead cryptococci adhere and are internalized by BMEC at the same rate as live cells. 1 ml aliquots of both encapsulated and non-capsulated cryptococci were heated at 65°C for 15 min prior to infection. Adherent and internalized yeasts were determined microscopically. To determine if the yeast culture was completely killed, 20 µl aliquots were plated on YPD agar and no growth was observed.

### Fixed endothelial cell control

As a negative control, mature endothelial cell (bEnd3 and hCMEC/D3) monolayers were fixed with 250 µl PFA 4% in PBS for 10 min at room temperature, rinsed five times with PBS and then inoculated with 2×10^6^ cryptococci per ml. As for the live endothelial cell monolayers, the fixed monolayers were incubated with cryptococci for 2 hr and 4 hr at 37°C with 5% CO_2_ and washed four times to remove non-adherent yeast cells. 200 µl sterile water was used to lyse the endothelial cells with additional scraping to remove any remaining endothelial cell associated cryptococci. The lysate was plated on YPD agar at 25°C and colony counts were made after 48 hr.

### Statistical analysis

Non-parametric Mann-Whitney U Test and Wilcoxon Signed Ranks Test were used to measure the significance of adherence at different conditions and time points. Mann-Whitney U Test was applied to compare the means of test different setups, for instance comparing the mean association of encapsulated and acapsular strain to bEnd3 and or hCMEC/D3 cells. Wilcoxon Signed ranks test was applied to compare means of the same setup at different time points, for example comparing the mean association of encapsulated H99 strain or the acapsular mutant with endothelial cells after 2 hrs and 4 hrs of incubation at 37°C.

## Results

### Cryptococcal association with and internalization by the murine brain endothelial cell line bEnd3

**Figure 1:**
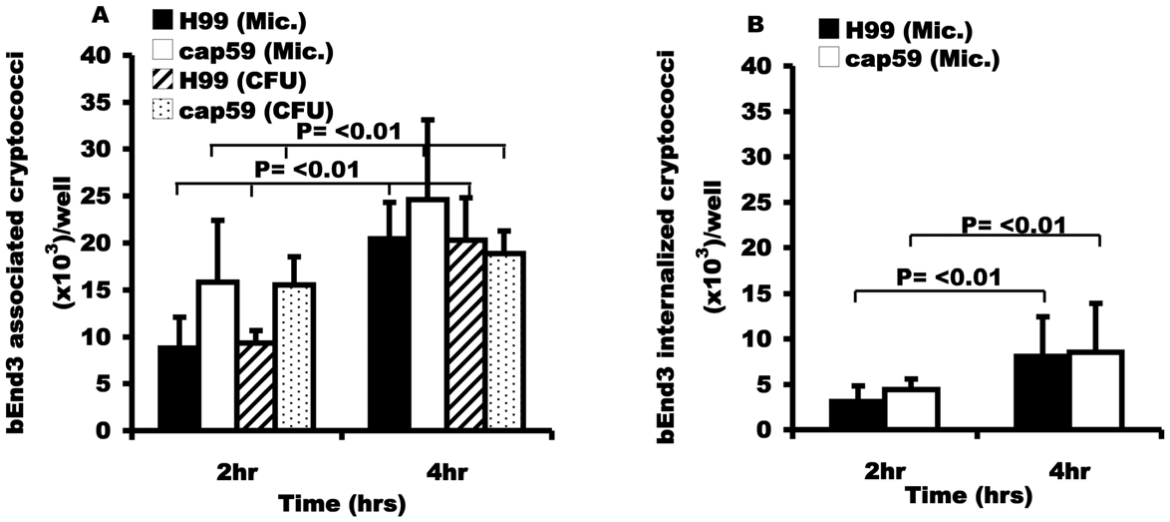
Binding and uptake of H99 and cap59 to the murine brain endothelial cell line, bEnd3. BEnd3 cells were exposed to wild type H99 and its acapsular derivative cap59 for 2 hr and 4 hr at 37°C. (A) The rate of association (bound and internalized cryptococci) was determined both by microscopy and live CFU counts. The rate of association increased significantly in a time dependent manner, (P<0.01 for both strains). However, there was no difference in association of encapsulated H99 or acapsular cap59 cells (P = 0.1 and 0.5 at 2 hr and 4 hr respectively). (B) Internalization of the two strains, H99 and cap59 by bEnd3 cells as determined by fluorescence microscopy. Internalized cryptococci (pre-stained with FITC) were distinguished from extracellular adherent ones by counter labelling with calcofluor white after infection. Intracellular cryptococci retained the FITC green signal while extracellular cells acquired the blue signal from calcofluor. The number of phagocytosed cryptococci increased significantly in a time dependent manner, (P<0.01 for both strains). However, there was no difference in internalization of encapsulated H99 and acapsular cap59, (P = 0.5 and 0.8 at 2 hr and 4 hr respectively). Error bars are standard error of the mean, n = 5 repeats.

We exposed the mouse brain endothelial cell line bEnd3 to wildtype C. *neoformans* H99 and its isogenic acapsular derivative, cap59 [38]. The two strains were tested for their rate of binding and internalization by BMEC and whether the presence or absence of capsule has an effect on this interaction. After two hours of exposure to bEnd3 cells at 37°C, 8.8×10^3^ (0.43%) of inoculated wild type (H99) cryptococci had adhered strongly to the endothelial layer, rising to 2.0×10^4^ (1.2%) after four hours. Similarly, 1.6×10^4^ (0.8%) of the acapsular cryptococci had associated with bEnd3 cells at 2 hr rising to 2.5×10^4^ (1.23%) after four hours of incubation at 37°C (Figure 1A). In contrast, binding to paraformaldehyde-fixed BMEC monolayers was negligible at all time points tested (Figure S1). By using calcoflour staining to discriminate surface bound from internalised cryptococci, we determined that 3.1×10^3^ (35% of associated) and 4.4×10^3^ (28% of associated) wild type and acapsular cryptococci, respectively, had been internalised by 2 hours at 37°C, rising to 8.1×10^3^ (40%) and 8.6×10^3^ (35%) respectively by 4 hrs (Figure 1B). Importantly, for both strains, microscopic yeast cell counts and live CFU counts gave the same rate of association for both H99 and cap59, suggesting that there is no significant drop in cryptococcal viability upon adherence to or uptake by bEnd3 cells.

### Effect of opsonisation on *Cryptococcus* – BMEC association and internalization

**Figure 2:**
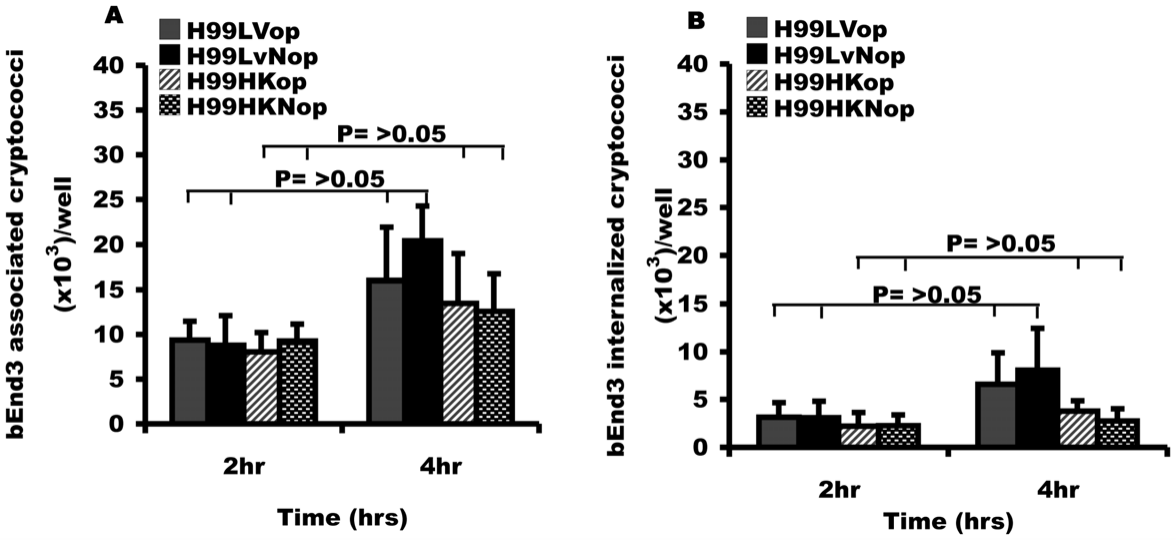
Effect of opsonisation on cryptococcal binding and uptake by bEnd3 cells. Live (LV) and heat-killed (HK) H99 cryptococci were opsonised with mouse derived anti-capsule IgG antibody, 18B7 and the rate of adherence and internalization was determined by fluorescence microscopy. (A) Association of opsonised (op) and non-opsonised (Nop) cryptococci with bEnd3 cells. Rate of association of opsonised and non-opsonised H99LV cryptococci was similar, P = 0.8 and 0.4 at 2 hr and 4 hr respectively. Similarly, there was no difference between opsonised and non-opsonised heat-killed H99 cryptococci, P = 0.9 and 0.5 at 2 hr and 4 hr respectively. (B) Rate of internalization of opsonised and non-opsonised cryptococci internalized by bEnd3 cells. The number of internalized H99LV cryptococci was similar, regardless of opsonisation status (P = 0.9 at both 2 hr and 4 hr). Similarly, there was no difference between opsonised and non-opsonised heat-killed H99 cryptococci, P = 0.5 and 0.4 at 2 hr and 4 hr respectively. Error bars are standard error of the mean, n = 5 repeats.

Since most individuals produce circulating antibodies to cryptococci by late childhood [34], we investigated whether opsonisation of cryptococci with antibody increases the association and internalization with brain endothelial cells. However, there were no significant differences between opsonised and non-opsonised yeast in either adherence or uptake at either time point tested (Figure 2A and B), suggesting binding and uptake by brain endothelial cells is opsonin independent.

### Cryptococcal association and internalization by human brain endothelial cells, hCMEC/D3

**Figure 3:**
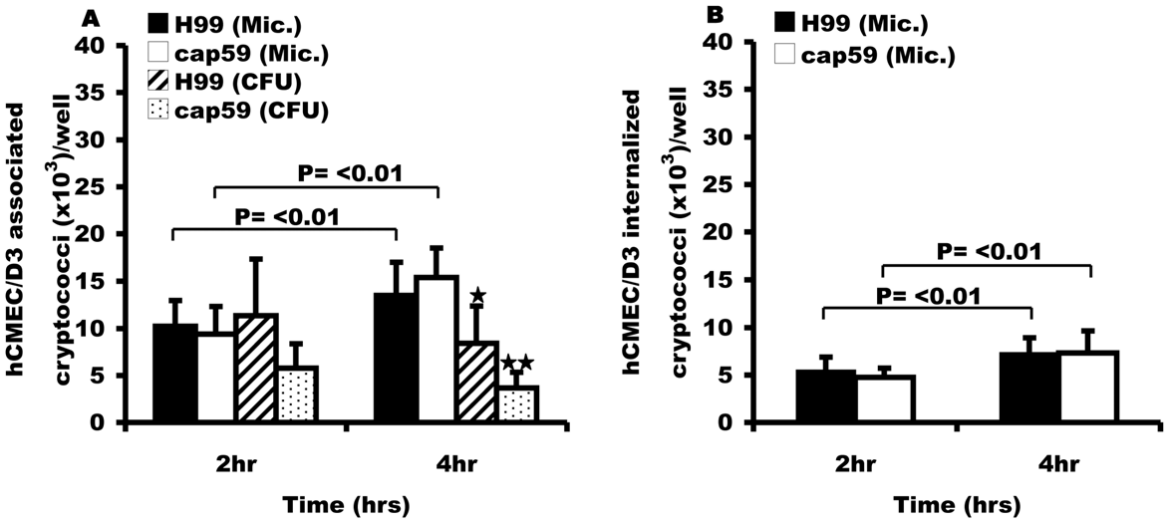
Binding and uptake of H99 and cap59 with human brain endothelial cell line, hCMEC/D3. Like bEnd3 cells, hCMEC/D3 cells were exposed to the *C. neoformans* serotype A isogenic strains, wild type H99 and acapsular derivative cap59 for 2 hr and 4 hr at 37°C. Microscopic (Mic.) and live CFU counts were performed to determine the association and survival of cryptococci. (A) Association efficiency of H99 and cap59 cryptococci with hCMEC/D3 cells. As opposed to microscopic counts, live CFU counts showed a time dependent decrease in the number of associated cryptococci by 4 hr of incubation, ★ = P<0.05 and ★★ = P<0.01 for H99 and cap59 respectively, suggesting a drop in viability of the hCMEC/D3 associated H99 and cap59 cryptococci. There was no difference in association of encapsulated H99 and acapsular cap59, P = 0.7 and 0.6 at 2 hr and 4 hr respectively. (B) Internalization of encapsulated H99 and acapsular cap59 cryptococci by human brain endothelial cell line, hCMEC/D3 cells. The internalized cryptococci and extracellular adherent were determined as in Figure 1. As with association, the number of phagocytosed cryptococci increased significantly in a time dependent manner, P = 0.01 and <0.01 for H99 and cap59 respectively. However, there was no difference in internalization of H99 and acapsular cap59 cryptococci, P = 0.5 and 0.8 at 2 hr and 4 hr respectively. Error bars are standard error of the mean, n = 6 repeats.

**Figure 4:**
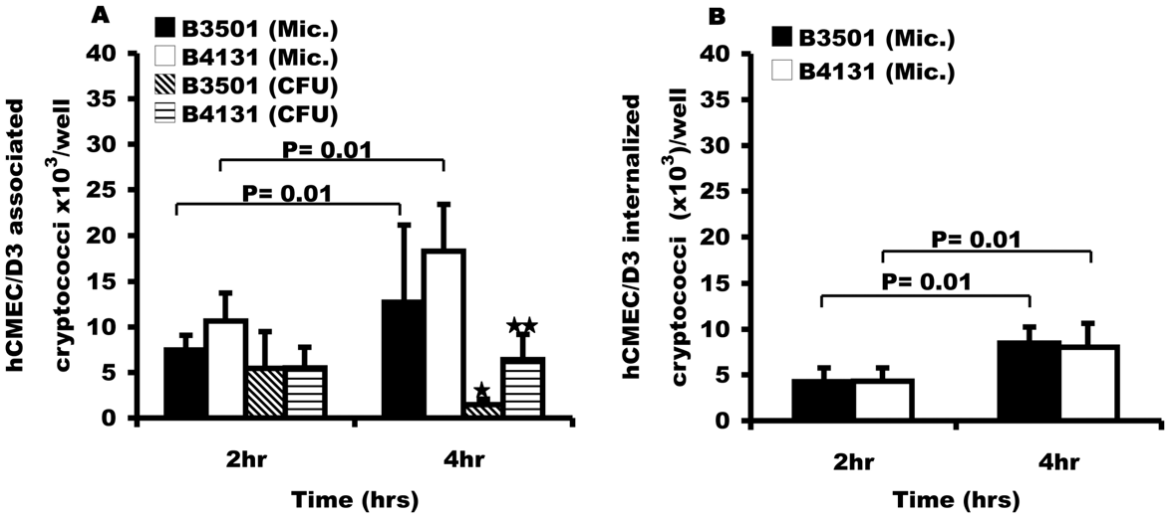
Binding and uptake of B3501 and B-4131 with human brain endothelial cell line, hCMEC/D3. HCMEC/D3 cells were exposed to *C. neoformans* serotype D wild type B3501 and its isogenic acapsular mutant B4131 for 2 hr and 4 hr at 37°C. Microscopic and live CFU counts were performed to determine the association and survival of cryptococci. (A) Association efficiency of B3501 and B4131 cryptococci with hCMEC/D3 cells. Like the H99-cap59 pair, live CFU counts showed a time dependent decrease in the number of associated cryptococci by 4 hr of incubation, ★ = P<0.01, ★★ = P<0.01 for both B3501 and B4131. There was no difference in association by encapsulated B3501 and acapsular B4131, P-value = >0.05 at 2 hr of incubation. However, B4131 was significantly more associated, P = 0.02 (Microscopy) and <0.01 (CFU) by 4 hr of incubation. (B) Internalization of encapsulated B3501 and its acapsular mutant derivative B4131 by hCMEC/D3 cells determined by fluorescence microscopy (Mic). The number of phagocytosed cryptococci increased significantly in a time dependent manner, P = 0.01 for both strains. However, there was no difference in internalization of B3501 and B4131, P = 0.5 and 0.2 at 2 hr and 4 hr respectively. Error bars are standard error of the mean, n = 6 repeats.

To test whether the rates of adherence and uptake that we had observed in bEnd3 cells were species specific, we repeated our analyses using the human brain endothelial derived cell-line, hCMEC/D3. As with bEnd3 cells, both microscopic and CFU counts showed that encapsulated H99 and its isogenic acapsular mutant cap59 associated and were engulfed at the same rate, P = >0.05 at 2 hr and 4 hr of infection respectively (Figure 3A and B). To determine whether the interaction varies from strain to strain, we tested a different pair of *C. neoformans* serotype D strains; encapsulated B3501 and its isogenic acapsular strain B4131. Like the H99/cap59 isogenic pair, both B3501 and B4131 strains associated and were internalized at the same rate with hCMEC/D3 cells (Figure 4A and B).

However, unlike bEnd3 cells, we observed a significant decrease over time in cryptococcal CFU counts for both strains. Microscopic counts revealed that association and internalization for both strains increases with time of incubation, suggesting a loss of viability by cryptococci during association with hCMEC/D3 cells (Figure 3A and 4A). One potential explanation for this result is that hCMEC/D3 cells may generate a more antimicrobial environment for cryptococci following uptake than that produced by bEnd3 cells. Thus bEnd3 cells and hCMEC/D3 cells bind and engulf cryptococci at similar rates, but only hCMEC/D3 cells are able to significantly reduce cryptococcal viability following uptake.

### Viability of cryptococci is not a prerequisite for association and internalization by BMEC

**Figure 5:**
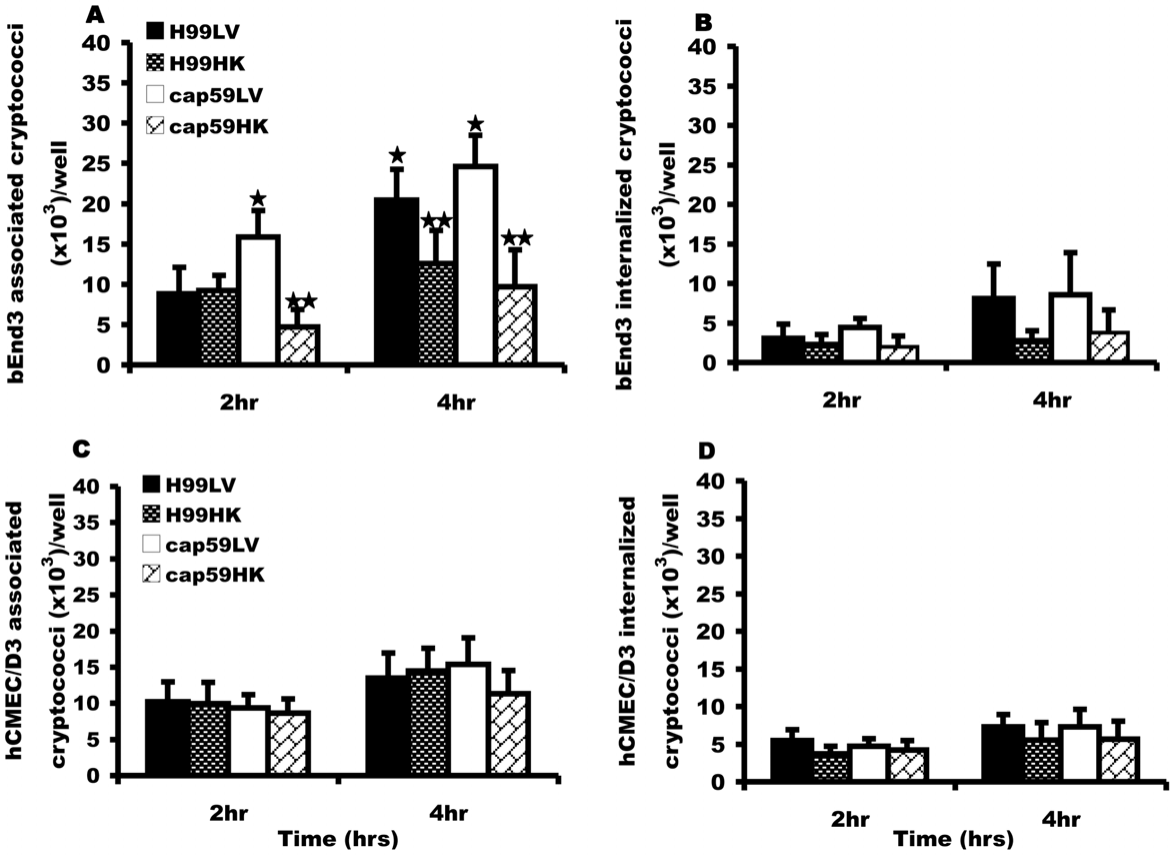
Effect of viability on binding and phagocytosis of H99 and cap59 strains by BMEC. Heating for 15 min at 65°C prior to infection killed H99 and cap59 cryptococci. Brain microvascular cells (BMEC), bEnd3 and or hCMEC/D3 cells were exposed in parallel to live (LV) and heat-killed (HK) cryptococci for 2 hr and 4 hr at 37°C and the number of bound and internalized cryptococci was determined by fluorescence microscopy. (A and C) Association efficiency of live and heat-killed H99 or cap59 cryptococci by bEnd3 and hCMEC/D3 cell respectively. There was no difference in association of viable H99 and cap59 cryptococci with hCMEC/D3 cells at either time point, P>0.05. However, viable cryptococci were more associated than non-viable ones by 4 hr of incubation in bEnd3 cells, P<0.01 for both H99 and cap59 respectively. (B and D) Internalization efficiency of LV and HK H99 and cap59 cryptococci by bEnd3 and hCMEC/D3 cells. Both live and heat-killed cryptococci showed a time dependent increase in phagocytosis by both bEnd3 and hCMEC/D3 cells, however the rate of phagocytosis did not vary between the viable and non-viable cryptococci in both cell-lines, P>0.05 at both 2 hr and 4 hr of incubation. Error bars are standard error of the mean, n = 5 repeats.

Lastly, we investigated whether the uptake of cryptococci into BMEC requires active signals from the pathogen. However, heat-killed H99 retained the ability to bind and enter both mouse bEnd3 and human hCMEC/D3 cells at a similar rate to live yeast (Figure 5A and C). Although viable cryptococci showed higher association efficiency in bEnd3 cells, there was no significant difference between internalization of viable and heat-killed cryptococci in both bEnd3 and hCMEC/D3 cells. (Figure 5B and D), suggesting that uptake is a passive process that occurs independently of yeast cell viability.

## Discussion

The mechanism by which *C. neoformans* associates with – and penetrates - the BBB remains a critical question in understanding the pathogenesis of CNS cryptococcosis. Considerable evidence shows that the penetration of the BBB by *C. neoformans* may occur via infected phagocytes [21], [39] or transcellularly through adherence and phagocytosis by the brain microvascular endothelial cells [22], [32]. Determining the relative contribution of these different routes to CNS cryptococcosis requires quantitative analysis of the interaction between cryptococci and brain microvascular cells, data that are currently lacking. Here we take the first steps to address this shortfall by using both mouse and human brain endothelial cell models.

Our data indicate that cryptococcal association with brain microvascular endothelial cells is a relatively infrequent event, although one that increases with extended periods of incubation. In both cell lines, mouse and human, less than 2% of encapsulated and acapsular cryptococci were strongly bound by 4 hrs of incubation. However, once bound, the probability of being internalized is high (typically >40% within four hours). These findings support recent observations in a mouse cryptococcosis model, which suggest that transmission across the BBB is a non-specific event dependent on trapping of cryptococci in narrow brain capillaries followed by phagocytosis into brain microvascular endothelial cells [32]. This implies that the adherence to – and phagocytosis of - cryptococci by BMEC is a slow process involving single cryptococci binding at any one time. We thus hypothesize that cryptococcal meningoencephalitis may be the result of a very small number of cryptococcal cells penetrating the BBB and subsequently proliferating to high numbers within the brain tissue. If so, then clinical approaches that reduce cryptococcal binding to endothelia even marginally may result in significant improvements to patient health. On the other hand, the low transcellular uptake suggests that *C. neoformans* might engage multiple entry mechanisms into the brain.

Possession of a capsule is a major virulence factor in *C. neoformans* and the presence of a capsule modulates many aspects of the interaction between cryptococci and infected hosts [40], [41]. *Cryptococcus* – endothelial cell interaction studies have yielded conflicting results regarding how capsule impacts on cryptococcal binding and uptake by BMEC [27], [29]–[31], [42]. By studying isogenic pairs of wild type and acapsular cryptococci and different brain endothelial cell-lines, we have shown that binding and uptake of cryptococci by BMEC is capsule independent. In agreement with this finding, intravital imaging of cryptococcal traversal of the blood-brain barrier has demonstrated equivalent crossing of the blood-brain barrier by wild type and acapsular cryptococcal yeast cells [32], an observation that implies the dispensability of the capsule for cryptococcal penetration of the blood-brain barrier.

Our finding that neither opsonisation nor active signalling from the cryptococcal cell are required for uptake are in line with recent data suggesting that cryptococcal uptake into brain endothelia is mediated by binding of endothelial CD44 to hyaluronic acid on the yeast surface [25]. Heat killing of the yeast is unlikely to disrupt this interaction. Although viable and non-viable (heat-killed) are equally internalized (this study), the non-viable cryptococci are unlikely to survive lysosomal degradation and hence may not transmigrate across the BBB in vivo [32]. Interestingly, however, our data suggest that human derived endothelia (but not murine bEnd3 cells) may be capable of killing intracellular cryptococci. Presumably crossing the BBB intact thus requires one or more of the virulence factors used by cryptococci to resist phagosomal killing [43], explaining why heat-killed cryptococci can enter endothelial cells but are not seen to transmigrate.

## Supporting Information

Figure S1 **Binding to fixed endothelial cell monolayers (negative control).** To verify that the observed binding occurred in association with endothelial cells, and not due to indirect sequestration of yeast cells, bEnd3 and hCMEC/D3 monolayers were killed by paraformaldehyde fixation prior to cryptococci inoculation. For all conditions, cryptococcal binding was reduced by between one and two log orders, indicating that that viable endothelial cells were responsible for the observed cryptococcal association.
(TIF)

## References

[1] Lin X (2009). *Cryptococcus neoformans*: Morphogenesis, infection, and evolution.. Infect Genet Evol.

[2] Park BJ, Wannemuehler KA, Marston BJ, Govender N, Pappas PG (2009). Estimation of the current global burden of cryptococcal meningitis among persons living with HIV/AIDS.. AIDS.

[3] Kidd SE, Hagen F, Tscharke RL, Huynh M, Bartlett KH (2004). A rare genotype of *Cryptococcus gattii* caused the cryptococcosis outbreak on vancouver island (british columbia, canada).. Proc Natl Acad Sci U S A.

[4] Byrnes EJ, Bildfell RJ, Frank SA, Mitchell TG, Marr KA (2009). Molecular evidence that the range of the Vancouver island outbreak of cryptococcus gattii infection has expanded into the pacific northwest in the united states.. J Infect Dis.

[5] Byrnes EJ, Li W, Lewit Y, Ma H, Voelz K (2010). Emergence and pathogenicity of highly virulent *Cryptococcus gattii* genotypes in the northwest united states.. PLoS Pathog.

[6] Byrnes EJ, Heitman J (2009). *Cryptococcus gattii* outbreak expands into the northwestern united states with fatal consequences.. F1000 Biol Rep.

[7] Datta K, Bartlett KH, Baer R, Byrnes E, Galanis E (2009). Spread of *Cryptococcus gattii* into pacific northwest region of the united states.. Emerging Infect Dis.

[8] Neofytos D, Fishman JA, Horn D, Anaissie E, Chang CH (2010). Epidemiology and outcome of invasive fungal infections in solid organ transplant recipients.. Transpl Infect Dis.

[9] Mitchell TG, Perfect JR (1995). Cryptococcosis in the era of AIDS–100 years after the discovery of *Cryptococcus neoformans*.. Clin Microbiol Rev.

[10] Huffnagle GB, McNeil LK (1999). Dissemination of *C. neoformans* to the central nervous system: Role of chemokines, Th1 immunity and leukocyte recruitment.. J Neurovirol.

[11] Casadevall A, Perfect JR (1998). *Cryptococcus neoformans*..

[12] Lortholary O (2007). Management of cryptococcal meningitis in AIDS: The need for specific studies in developing countries.. Clin Infect Dis.

[13] Boulware DR, Bonham SC, Meya DB, Wiesner DL, Park GS (2010). Paucity of initial cerebrospinal fluid inflammation in cryptococcal meningitis is associated with subsequent immune reconstitution inflammatory syndrome.. J Infect Dis.

[14] Nussbaum JC, Jackson A, Namarika D, Phulusa J, Kenala J (2010). Combination flucytosine and high-dose fluconazole compared with fluconazole monotherapy for the treatment of cryptococcal meningitis: A randomized trial in malawi.. Clin Infect Dis.

[15] Kambugu A, Meya DB, Rhein J, O'Brien M, Janoff EN (2008). Outcomes of cryptococcal meningitis in Uganda before and after the availability of highly active antiretroviral therapy.. Clin Infect Dis.

[16] Bicanic T, Harrison TS (2005). Cryptococcal meningitis.. Br Med Bull.

[17] Mwaba P, Mwansa J, Chintu C, Pobee J, Scarborough M (2001). Clinical presentation, natural history, and cumulative death rates of 230 adults with primary cryptococcal meningitis in zambian AIDS patients treated under local conditions..

[18] Kim KS (2008). Mechanisms of microbial traversal of the blood-brain barrier.. Nat Rev Microbiol.

[19] Brown RC, Morris AP, O'Neil RG (2007). Tight junction protein expression and barrier properties of immortalized mouse brain microvessel endothelial cells.. Brain Res.

[20] Correale J, Villa A (2009). Cellular elements of the blood-brain barrier..

[21] Charlier C, Nielsen K, Daou S, Brigitte M, Chretien F (2009). Evidence of a role for monocytes in dissemination and brain invasion by *Cryptococcus neoformans*.. Infect Immun.

[22] Chang YC, Stins MF, McCaffery MJ, Miller GF, Pare DR (2004). Cryptococcal yeast cells invade the central nervous system via transcellular penetration of the blood-brain barrier.. Infect Immun.

[23] Chang YC, Stins MF, McCaffery MJ, Miller GF, Pare DR (2004). Cryptococcal yeast cells invade the central nervous system via transcellular penetration of the blood-brain barrier.. Infect Immun.

[24] Eisenman HC, Casadevall A, McClelland EE (2007). New insights on the pathogenesis of invasive *Cryptococcus neoformans* infection.. Curr Infect Dis Rep.

[25] Huang SH, Long M, Wu CH, Kwon-Chung KJ, Chang YC (2011). Invasion of *Cryptococcus neoformans* into human brain microvascular endothelial cells is mediated through the lipid rafts-endocytic pathway via the dual specificity tyrosine phosphorylation-regulated kinase 3 (DYRK3).. J Biol Chem.

[26] Heitman J, Kozel TR, Kwon-Chung KJ, Perfect JR, Casadevall A, Washington (2010). *Cryptococcus*: From human pathogen to model yeast.. DC.

[27] Fries BC, Taborda CP, Serfass E, Casadevall A (2001). Phenotypic switching of *Cryptococcus neoformans* occurs in vivo and influences the outcome of infection.. J Clin Invest.

[28] Chen SH, Stins MF, Huang SH, Chen YH, Kwon-Chung K (2003). *Cryptococcus neoformans* induces alterations in the cytoskeleton of human brain microvascular endothelial cells.. J Med Microbiol.

[29] Charlier C, Chretien F, Baudrimont M, Mordelet E, Lortholary O (2005). Capsule structure changes associated with *Cryptococcus neoformans* crossing of the blood-brain barrier.. Am J Pathol.

[30] Guerrero A, Jain N, Goldman DL, Fries BC (2006). Phenotypic switching in *Cryptococcus neoformans*.. Microbiology.

[31] Jain N, Guerrero A, Fries BC (2006). Phenotypic switching and its implications for the pathogenesis of *Cryptococcus neoformans*.. FEMS Yeast Res.

[32] Shi M, Li SS, Zheng C, Jones GJ, Kim KS (2010). Real-time imaging of trapping and urease-dependent transmigration of *Cryptococcus neoformans* in mouse brain.. J Clin Invest.

[33] Jong AY, Stins MF, Huang SH, Chen SH, Kim KS (2001). Traversal of *Candida albicans* across human blood-brain barrier in vitro.. Infect Immun.

[34] Goldman DL, Khine H, Abadi J, Lindenberg DJ, Pirofski L (2001). Serologic evidence for *Cryptococcus neoformans* infection in early childhood.. Pediatrics.

[35] Mukherjee S, Lee SC, Casadevall A (1995). Antibodies to *Cryptococcus neoformans* glucuronoxylomannan enhance antifungal activity of murine macrophages.. Infect Immun.

[36] Vecchiarelli A, Pietrella D, Bistoni F, Kozel TR, Casadevall A (2002). Antibody to *Cryptococcus neoformans* capsular glucuronoxylomannan promotes expression of interleukin-12Rbeta2 subunit on human T cells in vitro through effects mediated by antigen-presenting cells.. Immunology.

[37] Bolanos B, Mitchell TG (1989). Phagocytosis and killing of *Cryptococcus neoformans* by rat alveolar macrophages in the absence of serum.. J Leukoc Biol.

[38] De Jesus M, Chow SK, Cordero RJ, Frases S, Casadevall A (2010). Galactoxylomannans from *Cryptococcus neoformans* varieties *neoformans* and *grubii* are structurally and antigenically variable.. Eukaryot Cell.

[39] Santangelo R, Zoellner H, Sorrell T, Wilson C, Donald C (2004). Role of extracellular phospholipases and mononuclear phagocytes in dissemination of cryptococcosis in a murine model.. Infect Immun.

[40] Syme RM, Bruno TF, Kozel TR, Mody CH (1999). The capsule of *Cryptococcus neoformans* reduces T-lymphocyte proliferation by reducing phagocytosis, which can be restored with anticapsular antibody.. Infect Immun.

[41] Zaragoza O, Chrisman CJ, Castelli MV, Frases S, Cuenca-Estrella M (2008). Capsule enlargement in *Cryptococcus neoformans* confers resistance to oxidative stress suggesting a mechanism for intracellular survival.. Cell Microbiol.

[42] Ibrahim AS, Filler SG, Alcouloumre MS, Kozel TR, Edwards JE (1995). Adherence to and damage of endothelial cells by *Cryptococcus neoformans* in vitro: Role of the capsule.. Infect Immun.

[43] Ma H, May RC (2009). Virulence in *Cryptococcus species*.. Adv Appl Microbiol.

