# Supplementary figures and images for: Capsule Independent Uptake of the Fungal Pathogen Cryptococcus neoformans into Brain Microvascular Endothelial Cells
Source: PLoS One. 2012 Apr 17;7(4):e35455. doi: 10.1371/journal.pone.0035455 (PMC3328471; doi:10.1371/journal.pone.0035455)

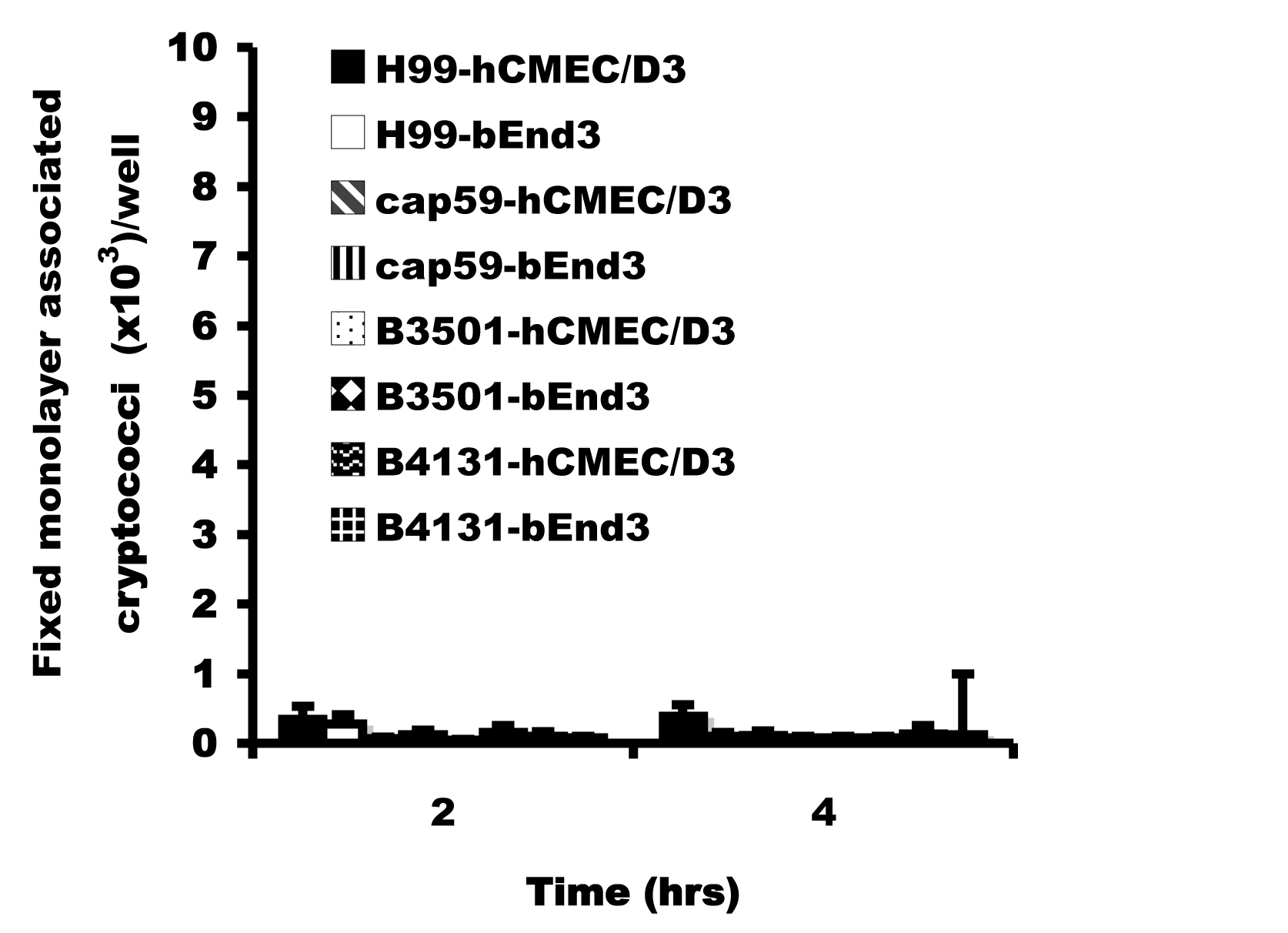

Supplement: Figure S1 — Binding to fixed endothelial cell monolayers (negative control). To verify that the observed binding occurred in association with endothelial cells, and not due to indirect sequestration of yeast cells, bEnd3 and hCMEC/D3 monolayers were killed by paraformaldehyde fixation prior to cryptococci inoculation. For all conditions, cryptococcal binding was reduced by between one and two log orders, indicating that that viable endothelial cells were responsible for the observed cryptococcal association. (TIF) [file pone.0035455.s001.tif]
